# Identification of a Benzamide Derivative that Inhibits Stress-Induced Adrenal Corticosteroid Synthesis 

**DOI:** 10.3390/molecules14093392

**Published:** 2009-09-03

**Authors:** Jing Xu, Laurent Lecanu, Matthew Tan, Janet Greeson, Vassilios Papadopoulos

**Affiliations:** 1 Department of Biochemistry & Molecular and Cellular Biology, Georgetown University Medical Center, Washington, DC 20057, USA; E-mails: jing@creatvmicrotech.com (J.X.); mt272@georgetown.edu (M.T.); 2 The Research Institute of the McGill University Health Centre, Montreal, Quebec, H3G 1A4, Canada; E-mail: laurent.lecanu@mcgill.ca (L.L.); 3 Department of Medicine, McGill University, Montreal, Quebec, H3G 1A4, Canada; 4 Samaritan Pharmaceuticals, Las Vegas, NV 89109, USA; E-mail: greesonjan@aol.com (J.G.); 5 Department of Pharmacology & Therapeutics, McGill University, Montreal, Quebec, H3G 1A4, Canada

**Keywords:** steroid synthesis, cholesterol uptake, cortisol, adrenal, neuroprotection

## Abstract

Elevated serum glucocorticoid levels contribute to the progression of many diseases, including depression, Alzheimer’s disease, hypertension, and acquired immunodeficiency syndrome. Here we show that the benzamide derivative *N*-[2-(4-cyclopropanecarbonyl-3-methyl-piperazin-1-yl)-1-(*tert*-butyl-1*H*-indol-3-yl-methyl)-2-oxo-ethyl]-4-nitrobenzamide (SP-10) inhibits dibutyryl cyclic AMP (dbcAMP)-induced corticosteroid synthesis in a dose-dependent manner in Y-1 adrenal cortical mouse tumor cells, without affecting basal steroid synthesis and reduced stress-induced corticosterone increases in rats without affecting the physiological levels of the steroid in blood. SP-10 did not affect cholesterol transport and metabolism by the mitochondria but was unexpectedly found to increase 3-hydroxy-3-methylglutaryl-coenzyme A, low density lipoprotein receptor, and scavenger receptor class B type I (SR-BI) expression. However, it also markedly reduced dbcAMP-induced NBD-cholesterol uptake, suggesting that this is a compensatory mechanism aimed at maintaining cholesterol levels. SP-10 also induced a redistribution of filamentous (F-) and monomeric (G-) actin, leading to decreased actin levels in the submembrane cytoskeleton suggesting that SP-10-induced changes in actin distribution might prevent the formation of microvilli– cellular structures required for SR-BI-mediated cholesterol uptake in adrenal cells.

## Abbreviations

ACTH: adrenocorticotropic hormone or corticotrophin
AD: Alzheimer’s disease
AIDS: acquired immunodeficiency syndrome
DAPI: 4',6'-diamidino-2-phenylindole hydrochloride
dbcAMP: dibutyryl cyclic AMP
F-actin: filamentous actin
G-actin: monomeric actin
HMGCR: 3-hydroxy-3-methylglutaryl-coenzyme A reductase
HPA: hypothalamus-pituitary-adrenal
LDL: low density lipoprotein
HDL: high density lipoprotein
MTT: 3-(4,5-dimethylthiazol-2-yl)-2,5-diphenyl tetrazolium bromide
NBD-cholesterol, 22-(*N*-(7-nitrobenz-2-oxa-1, 3- diazol-4-yl) amino)-23: 24-bisnor-5-cholen-3β-ol
P450scc: cytochrome P450 side chain cleavage (CYP11A1)
PBS: phosphate-buffered saline
PCR: polymerase chain reaction
Q-PCR: real-time quantitative PCR
SDS-PAGE: sodium dodecyl sulfate polyacrylamide gen electrophoresis
SP-10: N-[2-(4-cyclopropanecarbonyl-3-methyl-piperazin-1-yl)-1-(tert-butyl-1H-indol-3-yl-methyl)-2-oxo-ethyl]-4-nitro-benzamide
SR-BI: scavenger receptor class B type I
StAR: steroidogenesis acute regulatory protein
TSPO: translocator protein 18 kDa).

## 1. Introduction

Cortisol, the most abundant circulating glucocorticoid in humans, regulates the sleep cycle, metabolism, the immune system, mood, as well as memory and learning. Excessive cortisol synthesis leads to metabolic dysfunction, cognitive impairment [1] and immunosuppression [2]. Abnormalities in the hypothalamic-pituitary-adrenal (HPA) axis have been reported in psychiatric disorders, including depression and mood alterations [3,4], dementia [5,6,7], Alzheimer’s disease (AD) [8,9,10,11,12], acquired immunodeficiency syndrome (AIDS) [13,14,15] multiple sclerosis [16], and breast cancer [17]. It has been proposed that disruption of hormonal balance in these diseases leads to increased cortisol production and results in elevated cortisol concentrations in the cerebrospinal fluid [8,16], blood [10,12,14], urine [5], and saliva [11]. 

Cortisol, like all steroids, is derived from cholesterol. The conversion of cholesterol to pregnenolone, the precursor of steroid hormones, occurs in the mitochondria. The rate-limiting step is catalyzed by the first enzyme of the pathway, the cytochrome P450 side-chain cleavage enzyme complex (P450scc; CYP11A1), which catalyzes the irreversible cleavage of a 6-carbon residue from cholesterol [18]. Therefore, the mobilization of free cholesterol and its transport into the mitochondria are key steps for steroidogenesis. Hormones such as corticotrophin (ACTH), and its second messenger cAMP, accelerate these processes. The rapid release of ACTH from the pituitary in response to stress results in the acute stimulation of adrenal steroid synthesis, part of the body’s effort to adapt to the external stimuli. Although cholesterol transport into the mitochondria is the rate-determining step in steroid biosynthesis, steroid formation is also limited by the amount of available cholesterol. Cholesterol availability depends on its uptake by steroid-generating cells and on its rate of synthesis, where the determining factor is the rate-limiting enzyme 3-hydroxy-3-methylglutaryl-coenzyme A reductase (HMGCR) [19]. 

**Figure 1:**
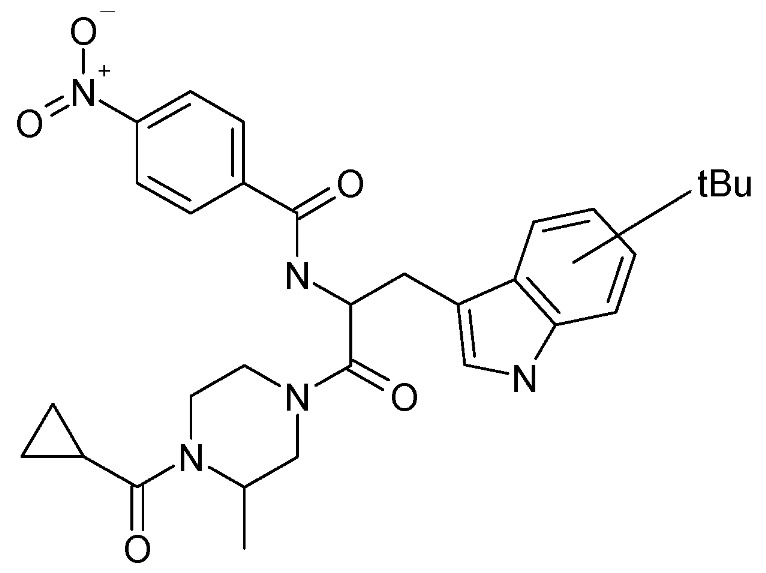
Chemical formula of SP-10, *N*-[2-(4-cyclopropanecarbonyl-3-methyl-piperazin-1-yl)-1-(*tert*-butyl-1*H*-indol-3-yl-methyl)-2-oxo-ethyl]-4-nitrobenzamide.

We recently demonstrated that both procaine and a procaine-based oral pharmaceutical formulations decreased both circulating glucocorticoid levels in rats and dbcAMP-induced steroid synthesis in adrenal cells by reducing both *hmgcr* mRNA expression and activity [20]. Here we provide both *in vitro* and *in vivo* evidence that *N*-[2-(4-cyclopropanecarbonyl-3-methyl-piperazin-1-yl)-1-(*tert*-butyl-1*H*-indol-3-yl-methyl)-2-oxo-ethyl]-4-nitrobenzamide (SP-10) (Figure 1), a benzamide derivative containing a stable procaine analog procainamide motif and that we previously described as having antiretroviral properties [21], decreases cholesterol uptake and steroidogenesis by altering actin filament formation and actin accumulation in the submembrane cytoskeleton. Thus, SP-10 by controlling the excessive formation of corticosteroids, known to lead to neurotoxicity, could offer a novel means of neuroprotection.

## 2. Results

### 2.1. SP-10 inhibits dbcAMP-induced steroid formation in Y-1 mouse adrenal cells

**Figure 2:**
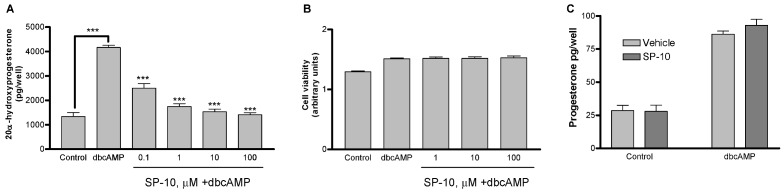
Effect of SP10 on the dbcAMP-induced 20α-hydroxyprogesterone synthesis and cell viability. Y-1 cells were incubated with the indicated concentrations of the SP-10 for 48 h. Culture medium was then changed and cultures were incubated with 1mM dbcAMP for an additional 48 h. (A) 20α-hydroxyprogesterone levels in the culture medium were determined by radioimmunoassay. (*** p < 0.001, ANOVA followed by Scheffe F test) (B) Alternatively, cell viability was measured using the MTT assay. (C) MA-10 Leydig cells were treated with dbcAMP ± 10 μM SP-10 using the same protocol as for Y-1 cells, and progesterone levels in culture media were determined. Results are presented as mean ± SD and represent three independent experiments performed in triplicate.

Levels of 20α-hydroxyprogesterone in Y-1 cultures treated with dbcAMP were approximately 4-fold higher than those in untreated cultures (Figure 2A; *p* < 0.001). Preincubation of the cells with SP-10 blocked dbcAMP-induced 20α-hydroxyprogesterone production in a dose-dependent manner (Figure 2A) with an IC_50_ value of 0.1 μM. Results shown in Figure 2A are from 48 h pre-incubation time period. The length of pre-incubation time and the efficacy of the treatment depended on the plating density and the achieved confluence after 24 or 48 h. Cells seeded at 40% confluence in 6-well plates reached a 70-80% confluence within 24 h whereas when seeded in 96-well plates reached 70-80% confluence in 48 h. Further treatment with dbcAMP brought cell confluence to 100%. SP-10 did not affect basal steroid formation (data not shown). SP-10 had no effect on Y-1 cell viability as determined by MTT assay (Figure 2B). In contrast to adrenal cells, dbcAMP-induced progesterone formation was not affected by SP-10 in MA-10 mouse Leydig tumor cells (Figure 2C). The viability of these cells was also unaffected by SP-10 (data not shown). 

### 2.2. SP-10 decreases circulating corticosterone levels in rats

To examine the *in vivo* effects of SP-10, rats were treated daily for 10 consecutive days with varying concentrations of SP-10 (1, 3, or 10 mg/kg), and serum corticosterone levels were determined 24 hours after the final treatment. 

**Figure 3:**
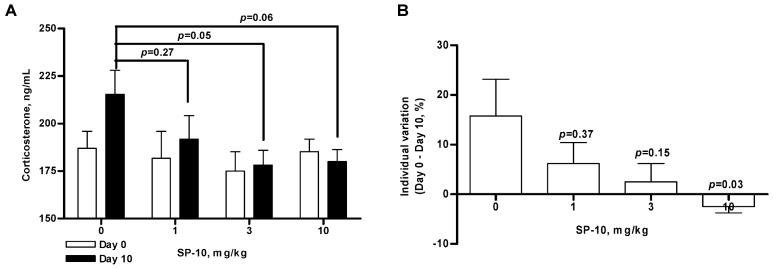
Male Long-Evans rats weighing 300-350 g were treated with the indicated concentrations of SP-10 once daily (i.p.) for 10 consecutive days. Blood (400 μL) was collected prior to dosing (Day 0) and 24 hours after the final dose (Day 10). Serum corticosterone levels were measured by radioimunoassay. (A) Dose response effect of SP-10 on Day 0 and Day 10 corticosterone concentrations. (B) Data in (A) are presented as the mean percent change in corticosterone levels from Day 0 to Day 10 to better illustrate the dose-dependent effects of SP-10. (ANOVA followed by Scheffe F test, n = 5 per group).

There were no differences in the basal corticosterone serum levels between each group (Figure 3A, n = 5). Treatment for 10 days with vehicle resulted in a 15.8±16.5% increase in corticosterone concentrations - as calculated by the mean percent change between pre-dose (day 0) and day 10 concentrations (Figure 3B). This increase was most likely due to the handling-related stress. SP-10 treatment reduced the amplitude of this corticosterone increase, although the effect was statistically significant for only the 3 mg/kg dose (p = 0.05, n = 5). Doses of 10 mg/kg had effects at the limit of the significance (p = 0.06, n = 5). However, a comparison of individual variations between day 0 and day 10 revealed that SP-10 significantly reduced stress-related corticosterone increases in a dose-dependent manner (Figure 3B), with the most pronounced effects being observed with the 10 mg/kg dose (versus control, p = 0.03, n = 5). Interestingly, the highest doses of SP-10 employed did not affect basal serum corticosterone levels. 

### 2.3. Effects of SP-10 on the steroidogenic pathway

We measured P450scc activity by using 22*R*-hydroxycholesterol, a hydrosoluble cholesterol derivative that can freely cross mitochondrial membranes and is a direct substrate of P450scc [18]. 

**Figure 4:**
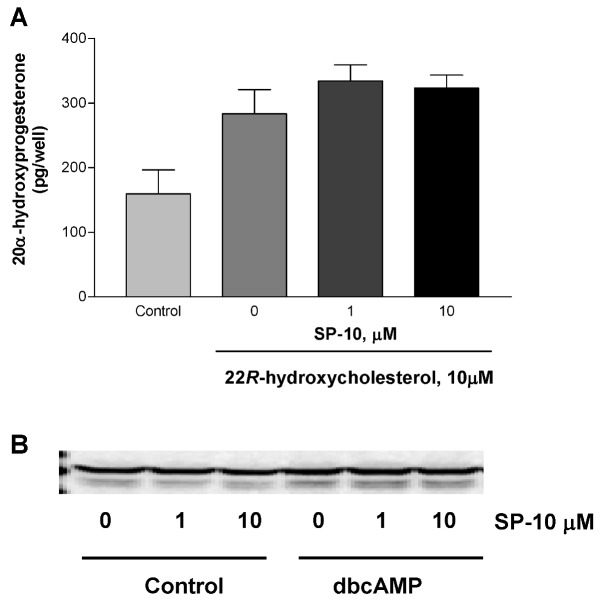
Effect of SP-10 on the cytochrome P450scc activity and P450scc protein expression in Y-1 cells. (A) Y-1 cells were treated with indicated concentrations of SP-10 for 24 h and then treated with 10 μM 22*R*-hydroxycholesterol for an additional 24 h. 20α-hydroxyprogesterone levels were measured in the medium by radioimmunoassay. Results are presented as mean ± SD and represent three independent experiments performed in triplicate. The effect of the 22*R*-hydroxycholesterol on steroid formation was statistically significant (p < 0.02, ANOVA). (B) Y-1 cells were treated with the indicated concentrations of SP-10 for 24 h, followed by treatment with 1mM dbcAMP for 24 h. P450scc protein levels were then determined by immunoblot analysis.

As expected, 22*R*-hydroxycholesterol treatment was associated with increased 20α-hydroxyprogesterone levels compared to vehicle-treated Y-1 cells (Figure 4A). However, this increase in 20α-hydroxyprogesterone was not affected by SP-10 (Figure 4A). In addition, SP-10 did not alter P450scc enzyme levels in cells treated with or without dbcAMP as assessed by immunoblot analysis (Figure 4B). These data suggest that enzymes involved in the conversion of cholesterol to 20α-hydroxyprogesterone were not affected by SP-10 treatment. 

**Figure 5:**
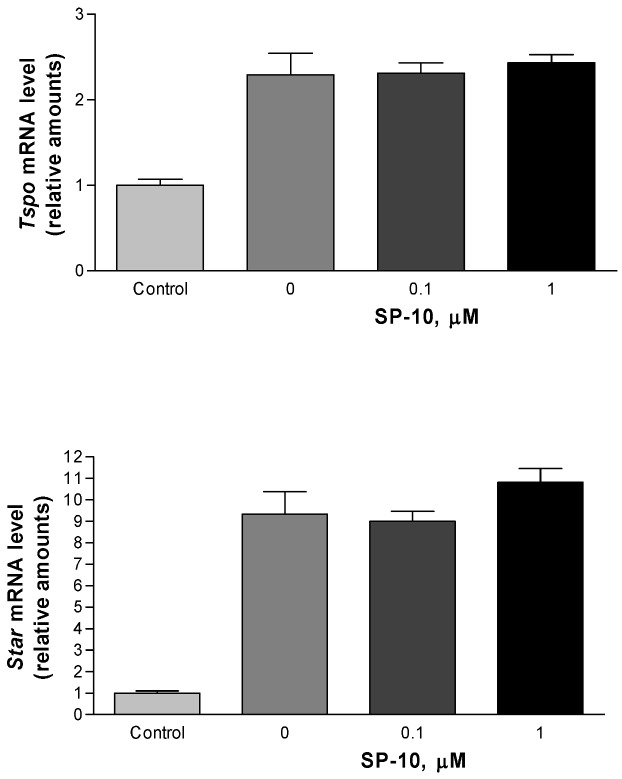
Effect of SP-10 on *Tspo* and *Star* mRNA levels. Y-1 cell were treated with the indicated concentrations of SP-10 for 24 h. This was followed by treatment with 1mM dbcAMP for 24 h. *Tspo* and *Sta* mRNA levels were quantified by Q-PCR. Results are presented as mean ± SD (n = 9 for each group). *Tspo* and *Star* mRNA levels were significantly increased by dbcAMP (p < 0.05, ANOVA).

Since the transport of free cholesterol from intracellular stores into mitochondria is the rate-determining step in steroid biosynthesis, we examined the dose-dependent effects of SP-10 on the mRNA levels of the translocator protein (18 kDa), previously know as the peripheral;-type benzodiazepine receptor, and the steroidogenesis acute regulatory protein (StAR), two key regulatory proteins that mediate the transfer of cholesterol into mitochondria [22,23]. The results revealed that24 h of dbcAMP treatment increased *Tspo* and *Star* mRNA by 2.3-fold and 9-fold, respectively (Figure 5A and B). Pre-treatment with increasing concentrations of SP-10 prior to dbcAMP treatment did not alter *Tspo* and *Star* mRNA levels (Figure 5A and B). SP-10 also did not affect the ligand binding characteristics of TSPO (data not shown). 

### 2.4. SP-10 increases hmgcr mRNA expression

**Figure 6:**
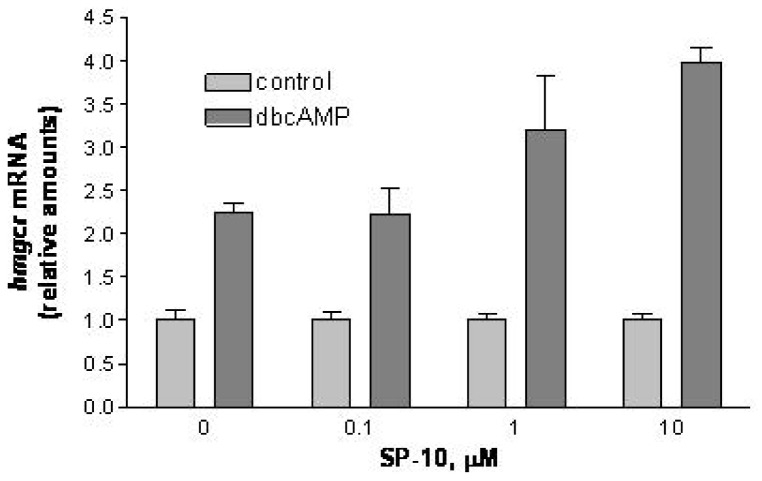
Effect of SP-10 on *hmgcr* mRNA levels. Y-1 cells were treated with the indicated concentrations of SP-10 for 24 h. Cultures were then treated with 1mM dbcAMP for an additional 24 h. *hmgcr* mRNA was quantified by Q-PCR using 18SRNA as an internal standard. Results are presented as mean ± SD and represent three independent experiments performed in triplicate. SP-10 at concentrations of 10 μM significantly enhanced dbcAMP-induced increases in *hmgcr* mRNA levels. (p < 0.05, ANOVA).

The lack of effect of SP-10 on the critical steps of the steroidogenic pathway suggested that this compound may exert its effects by altering the amount of the cholesterol substrate available for transport and metabolism. Therefore, we examined the effect of SP-10 on dbcAMP-induced increases in *hmgcr* mRNA levels. As shown in Figure 6, pre-treatment of Y-1 cells with SP-10 for 24 h enhanced dbcAMP-induced increases in *hmgcr* mRNA levels in a dose-dependent manner (*p* < 0.05, ANOVA). 

### 2.5. SP-10 inhibits NBD-cholesterol uptake

**Figure 7:**
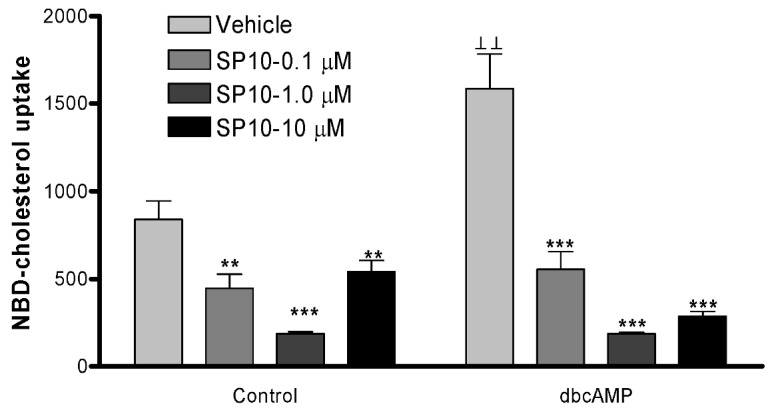
Effect of SP-10 on NBD-cholesterol uptake. Y-1 cells were incubated with the indicated concentrations of SP-10 for 48 h. Culture media were then changed, and cultures were incubated with 1mM dbcAMP and 1μg/ml NBD-cholesterol for 24 h. NBD-cholesterol uptake was measured by a Vector^2^ multilable counter equipped with 485 nm excitation and 535 nm emission filters. Total fluorescence values were normalized to cellular protein. Results are presented as mean ± SD (n = 12 for each group). (** p < 0.01, *** p < 0.001 versus vehicle-treated group, ┴┴ p < 0.01 versus vehicle-treated control group, ANOVA followed by Scheffe F test).

Since SP-10 did not inhibit dbcAMP-stimulated increases in *hmgcr* mRNA levels, we examined its effect on cholesterol uptake. To do this, we used the fluorescent cholesterol analog, NBD-cholesterol, as a probe for monitoring cholesterol flux [24,25]. Treatment of Y-1 cells with dbcAMP increased the uptake of NBD-cholesterol by 2-fold (Figure 7). Pretreatment of cells with SP-10 reduced both basal and dbcAMP-induced NBD-cholesterol uptake (Figure 7). SP-10 exerted a significant effect at concentrations as low as 0.1 μM where it inhibited dbcAMP-induced NBD-cholesterol uptake by 60% (*p* < 0.001).

### 2.6. Effects of SP-10 on LDL receptor and SR-BI receptor expression

LDL and HDL cholesterol are the major sources of cholesterol for steroid synthesis [26]. LDL cholesterol uptake is mediated by LDL receptor-mediated endocytosis and HDL cholesterol uptake is mediated by the SR-BI receptor. 

**Figure 8:**
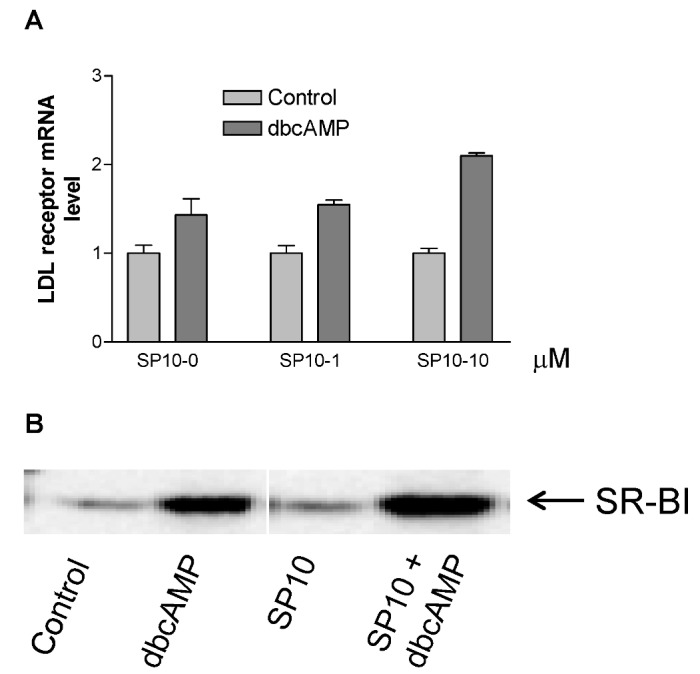
Effect of SP-10 on LDL-R mRNA levels and SR-BI protein levels. Y-1 cells were treated with the indicated concentration of SP10 for 24 h. They were then treated with 1 mM dbcAMP for 24 h. (A) LDL-R mRNA was quantified by Q-PCR. Results are presented as mean ± SD. (n ≥ 3 for each group). SP-10 at concentrations of 10μM significantly enhanced dbcAMP-induced increases in LDL-R mRNA levels (p < 0.01, ANOVA). **B.** SR-BI receptor protein levels were determined by immunoblot analysis.

Since SP-10 inhibited NBD-cholesterol uptake, we examined its effect on LDL receptor mRNA levels and SR-BI receptor protein levels. DbcAMP increased total cellular LDL receptor mRNA levels compared with control, although the increase seen was not statistically significant (Figure 8A). However, pretreatment of cells with SP-10 significantly increased the dbcAMP-stimulated LDL receptor mRNA expression in Y-1 cells. In the same way, SP-10 (10 μM) also enhanced dbcAMP-induced increases in SR-BI protein levels (Figure 8B).

### 2.7. SP-10 alters actin cytoskeleton dynamics in Y-1 cells

**Figure 9:**
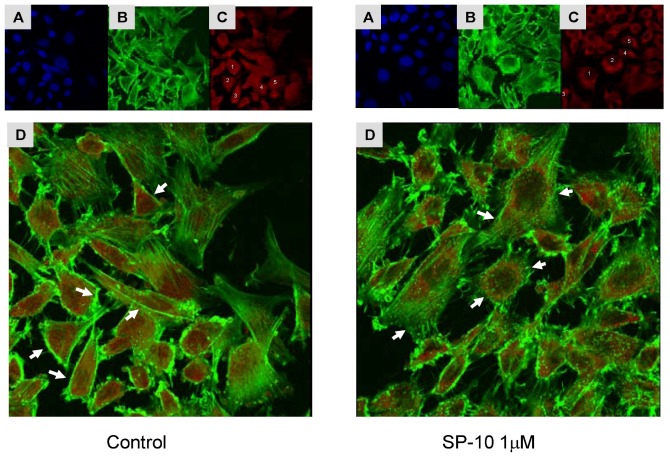
Effect of SP-10 on actin localization. Y-1 cells grown on coverslips were treated with SP-10 for 48 h, then washed with PBS and fixed in 4% paraformaldehyde. The cell membranes were permeabilized with 0.1% Triton X-100, then stained with Alexa Fluor 488 phalloidin for F-actin (Green) and Alexa Fluor 594 Deoxyribonuclease I (red) for G-actin. Nuclear DAPI staining (blue; A), F-actin (B) and G-actin (C) localization in control and SP-10 treated cells are shown in the top panels. Panel D shows merged images of F- and G- actin localization. Arrows indicate changes in F-actin distribution. Cells numbered from 1 to 5 on panel C were used for F-actin and G-actin fluorescence quantification.

**Figure 10:**
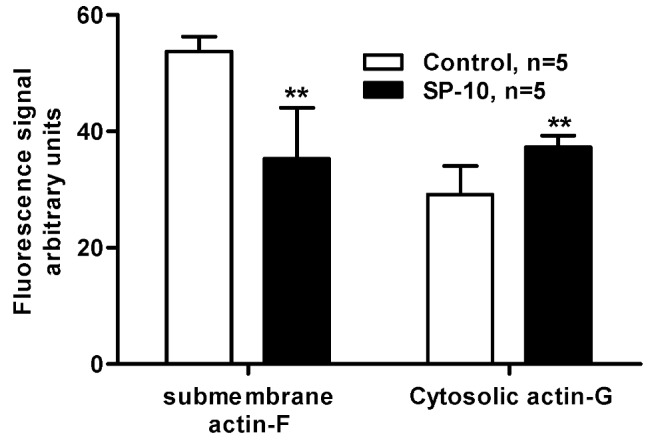
Submembrane F-actin and cytosolic G-actin quantification. The quantification was performed using NIG ImageJ software on five representative cells of each group, control and SP-10-treated, as numbered on Figure 9 panel C. Results are presented as means ± SD (n = 5 for each group). (** p < 0.01 versus control group, t-test).

Phalloidin tightly binds to F-actin filaments but not to the G-actin monomers. Therefore, the amount of bound phalloidin reflects the amount of actin filaments, and F-actin can be measured using a fluorescent derivative of phalloidin [27,28,29]. As shown in Figure 9, confocal microscopy of Alexa Fluor 488-phalloidin labeling revealed that treatment of Y-1 cells with SP-10 decreased the amount of F-actin present in the cytosol. It also decreased the amount of F-actin close to the cell membrane and a moderate increase in G- actin was seen in the cells. The alteration of F-actin and G-actin levels was further quantified using the NIH ImageJ software (Figure 10). These data suggest that SP-10 induces changes in actin distribution, a finding supported by the fact that the amount of total actin in the cells was not affected by the treatment as shown by immunoblot analysis (data no shown).

## 3. Discussion

Y-1 mouse adrenal tumor cells have been extensively used to study adrenal steroid formation. 20α-hydroxyprogesterone, which results from the conversion of progesterone by 20α-hydroxylase, is the major steroid formed in these cells [30]. Here we demonstrate that the benzamide derivative SP-10 inhibits the dbcAMP-induced 20α-hydroxyprogesterone production in Y-1 cells in a dose-dependent manner. Interestingly, SP-10 does not affect basal steroid formation, suggesting that it exerts its modulatory activity only in the presence of a stimulus. As an *in vivo* correlate, we assessed the ability of SP-10 to modulate corticosterone synthesis in Long-Evans rats. Long-Evans rats were chosen since this strain develops an Alzheimer’s disease-like phenotype under pharmacological pressure [31]. Interestingly, high cortisol serum levels, that are non-responsive to dexamethasone, have been reported in Alzheimer’s patients [32]. Animals receiving vehicle for ten days exhibited increased serum corticosterone concentrations, likely due to handling-related stress. SP-10 blocked stress-induced corticosterone increases in a dose-dependent manner, with 3 or 10 mg/kg doses returning corticosterone levels to baseline. This dose-effect relationship was more apparent when the mean percent change between day 0 and day 10 levels were compared. As was the case *in vitro*, SP-10 did not alter basal corticosterone concentrations, confirming that SP-10 only modulates corticosterone levels in the presence of hormonal stimuli/stressors. Importantly, these effects are tissue-specific, as SP-10 did not affect cAMP-induced steroid formation in a mouse Leydig cell line. In support of this finding, the tissue-specific transcriptional regulation of key protein components of the steroidogenic pathway has been demonstrated [33]. Nevertheless, this modulatory effect of SP-10 on adrenal cells remains to be assessed in brain and ovarian steroid-synthesizing cells. It should also be noted that under our experimental conditions, SP-10 did not affect cell viability. Taken together, these results suggest that SP-10 may be good lead candidate for targeting pathologies associated with increased activity of the HPA axis and subsequent high cortisol production. 

To understand the mechanism by which SP-10 blocks cAMP-induced adrenal steroidogenesis, we first examined the effects of SP-10 on the steroidogenic pathway. SP-10 did not affect the rate of steroid formation in Y-1 cells incubated in the presence of 22*R*-hydroxycholesterol, a substrate of the P450scc enzyme, suggesting that the enzymes involved in the formation of the steroid end products are unaffected by this treatment. This result was further supported by the finding that P450scc protein expression was unaffected by SP-10. In addition, SP-10 did not affect mRNA levels of *Tspo* and *Star*, two key regulatory proteins that mediate the transfer of cholesterol into the mitochondria [22,23]. Based on these data, we examined the effects of SP-10 on upstream events. Treatment of Y-1 cells with dbcAMP resulted in increased *hmgcr* mRNA levels. Surprisingly, inclusion of SP-10 enhanced these increases. These data suggest that SP-10 does not decrease the *de novo* synthesis of cholesterol by adrenal cells and it does not interfere with the cholesterol transfer into mitochondria necessary for steroid synthesis. Thus, the inhibitory effect of SP-10 may be located further upstream in the pathway. 

Next, we tested the hypothesis that SP-10 affects the uptake of cholesterol by monitoring NBD-cholesterol uptake in adrenal cells. The results revealed that SP-10 did reduce the uptake of NBD-cholesterol by Y-1 cells, and it did so regardless of whether cAMP was present. 

Cholesterol uptake in mammalian cells occurs via one of two pathways depending on the nature of the lipoprotein and the function of the cell [19,34]. LDL receptor mediates cholesterol uptake from LDL and SR-BI mediates cholesterol uptakes from HDL. LDL receptor is a high-affinity/low-capacity receptor whereas SR-BI is a low affinity/ high capacity receptor. Although both receptors are present in mammalian steroidogenic cells, the latter accounts for the majority of HDL-cholesterol uptake, particularly in the adrenal cells, since steroid synthesis requires large amounts of cholesterol [19,35]. In agreement with previous reports, dbcAMP increased the expression of both LDL-R and SR-BI in adrenal cells [19,36]. Although SP-10 did not exert any effects when added to the cells alone, it increased dbc-AMP-induced LDL receptor and SR-BI expression. These results were surprising because we showed that SP-10 inhibits cholesterol uptake. However, two previous studies reported that treating rats and mice with the hypocholesterolemic agent, 17α-ethinylestradiol, also resulted in an increase in SR-BI expression [37,38]. This effect was attributed to an up-regulation of receptor expression consequent to low cholesterol serum concentrations. Interestingly, this up-regulation appears to be organ-specific because it was documented in the testis, another steroidogenic organ, but not in the liver [39]. In contrast, the broadly used hypocholesterolemic class of fibrates was shown to increase SR-BI expression in the liver but not in the adrenals of mice [39,40], challenging the idea that hypocholesterolemia triggers SR-BI up-regulation in adrenal glands..

The mechanism by which SR-BI internalizes free cholesterol and cholesteryl esters in the adrenal cells is still poorly understood. Earlier studies demonstrated that SR-BI was present in caveolae where it was co-localized with caveolin [34]. This finding could explain, at least in part, the mechanism by which adrenal cells internalize cholesterol following an interaction between SR-BI and apoliporpotein-A1. However, studies in human adrenal cells revealed that the cells do not have caveolae and that there is no co-localization between SR-BI and caveolin [41]. It was recently reported that ACTH induces SR-BI dimerization and oligomer formation, leading to an increase in [^125^I/^3^H]hHDL_3_ cholesterol uptake [42]. It has also been shown that ACTH-induced corticosterone increases in rats are associated with SR-BI dimerization/oligomerization as well as a striking modification of the adrenal cell architecture, leading to the formation of microvilli, intracellular channels, and the re-localization of the SR-BI dimers and oligomers in these cellular structures [38]. 

Actin, one of the most ubiquitous and evolutionarily conserved structural proteins and a major component of the mammalian cytoskeleton, plays an essential role to form and maintain cell shape and structure [43]. Y-1 cells round up quite dramatically when exposed to dbcAMP, an event accompanied by actin accumulation near the cell membrane, filament formation [43], and increased steroidogenesis [44,45]. Indeed, ACTH treatment has been shown to modify F-actin localization in rat adrenal cells [46,47]. Consistent with these findings, we have observed that SP-10 alters the distribution of F- and G-actin such that the amount of actin in the submembrane cytoskeleton is diminished, and the amount of actin in the internal cytosol is enriched. Interestingly, it has been reported that changes in the distribution and pools of G- and F-actin affects the hormone-induced steroid formation in adrenocortical cells [48]. Based on this data, we hypothesize that SP-10 blocks cholesterol uptake in adrenal cells by modifying actin dynamics, thereby leading to a reduction of microvilli formation and a re-localization of SR-BI. This hypothesis awaits further testing although the data presented herein confirm and extend recent findings indicating that SP-10 exerts antiretroviral properties by blocking HIV-1 entry in the host cell through an alteration of the actin polymerization/depolymerization dynamic [21]. SP-10 was found to alter both basal and cAMP-induced cholesterol uptake, although it did not affect basal steroid production. These findings suggest that under basal conditions there is enough cholesterol in the cells to sustain steroid formation.

## 4. Conclusions

In conclusion, we have presented evidence that the benzamide derivative, SP-10, reduces hormone and stressor-stimulated glucocorticoid synthesis, both *in vitro* and *in vivo*, by reducing cholesterol uptake by adrenal cells and thus, the amount of cholesterol available for steroidogenesis. Although not fully understood, SP-10 effects might be attributable to a modification in actin dynamics and subsequent prevention of the formation of microvilli− sites of SR-BI dimer and oligomer translocation. Elevated cortisol concentrations are associated with many diseases and are known to worsen the outcomes of these conditions. Although high levels of cortisol are clearly detrimental, maintenance of basal cortisol levels is necessary for basic biological functions. 

SP-10 did not affect basal corticosteroid production. Development of compounds which block excessive glucocorticoid synthesis without affecting basal steroid formation could be valuable, either as monotherapies or complementary therapies, for the treatment of diseases associated with high cortisol levels such as, AIDS and HIV-associated cognitive impairment, Cushing syndrome (a neuropsychiatric disorder), depression, multiple sclerosis, and AD [3,4,5,6,7,8,9,10,11,12,13,14,15,16,52,53]. Considering the ability of SP-10 to block the stress-induced excessive formation of corticosteroids, that affect the metabolic, cardiovascular and central nervous system, and known to lead to neuroendangerment and ultimately neurotoxicity, could offer a novel means of neuroprotection.

## 5. Experimental

### 5.1. Materials

Y-1 mouse adrenal tumor cell line, F-12K (Kaign’s modification of Ham’s F-12) medium, DMEM/Ham’s F-12 medium, horse serum, and fetal bovine serum (FBS) were purchased from the American Type Culture Collection (Manassas, VA, USA). The MA-10 mouse Leydig tumor cell line was generously provided by Dr. Mario Ascoli (University of Iowa, IA, USA). The synthesis and characterization of SP-10 were recently reported [21]. Fetal bovine lipoprotein deficient serum was obtained from Intracel Corporation (Frederick, MD, USA) and the protease inhibitor cocktail was from Sigma (St. Louis, MO, USA). Anti-20α-hydroxyprogesterone antibody was purchased from Endocrine Sciences (Calabasas, CA, USA). Anti-progesterone and anti-corticosterone antibodies were purchased from ICN Pharmaceuticals (Costa Mesa, CA, USA). Anti-P450scc antibody was obtained from Research Diagnostics Inc. (Flanders, NJ, USA), anti-αactin antibody was from Sigma and anti-SR-BI receptor was from Abcam (Cambridgeshire, UK). ^3^H-20α-hydroxyprogesterone was from American Radiolabeled Chemical, Inc. (St. Louis, MO, USA). ^3^H-progesterone, 1,2,6,7-^3^H (N)-corticosterone, and ^3^H-pregnenolone were purchased from PerkinElmer Life Sciences Inc. (Boston, MA, USA). The MTT cell proliferation assay kit was obtained from Trevigen, Inc. (Gaithersburg, MD, USA). RNA STAT-60 was purchased from TEL-TEST, Inc. (Friendswood, TX, USA) and TaqMan® Reverse Transcription Reagents, random hexamers, and SYBR® Green PCR Master Mix were purchased from Applied Biosystems (Foster City, CA, USA). Alexa Fluor 488 phalloidin, Alexa Fluor 594 Deoxyribonuclease I, and 22-(*N*-(7-nitrobenz-2-oxa-1,3-diazol-4-yl)amino)-23,24-bisnor-5-cholen-3β-ol (NBD-cholesterol) were from Molecular Probes, Inc. (Eugene, OR, USA)**.** Cell culture plasticware was from BD Falcon (Franklin Lakes, NJ, USA). 

### 5.2. Animal treatment

Male Long-Evans rats, weighing 300–350 g at time of dosing, were purchased from Charles River Breeding Laboratories (Wilmington, MA, USA). All animals were housed at the Georgetown University Research Resources Facility under controlled light and temperature. Food and water was allowed *ad libitum*. All experimental protocols were reviewed and approved by the Georgetown University Animal Care and Use Committee. SP-10 (1, 3, and 10 mg/kg) and vehicle (1% ethanol in 0.9% NaCl) were intraperitoneally (i.p.) administered once daily for 10 consecutive days. Blood (400 μl) was collected prior to dosing and 24 h after the final dosing through the tail vein in rats deeply anesthetized with Equitesin,1 mL/300g. Corticosterone ratios were calculated as means of animal individual ratios for each group. Each animal was handled for a maximum of five minutes between the first handling for anesthetic injection and the end of the blood draw. All the animals were bled within the same two hours period in the morning at Day 0 and at Day 10. In addition all the animals were handled identically. Each individual ratio was calculated using corticosterone values measured on blood samples collected at the same period of the day. In our experimental conditions, the only variable was the SP-10 dose that was administered. Corticosterone levels were determined by radioimmunoassay (RIA) as previously described [20].

### 5.3. Cell culture

Y-1 mouse adrenal tumor cells were maintained in F12K medium containing 15% FBS under a 5% CO_2_ atmosphere. MA-10 mouse Leydig tumor cells were maintained in DMEM/F12 medium supplemented with 5% FBS and 2.5% horse serum under a 4% CO_2_ atmosphere [30].

### 5.4. Determination of steroid synthesis

Y-1 or MA-10 cells were cultured in 96-well plates (2 x 10^4^cells/well). Forty-eight hours later, cells were treated with increasing concentrations of SP-10 for 24 to 48 h. Culture media were then changed and cells were stimulated with 1 mM dbcAMP for 24 to 48 h. Synthesis of 20α-hydroxyprogesterone and progesterone in Y-1 and MA-10 cells, respectively, was measured by radioimmunoassay [30]. 

### 5.5. Analysis of mitochondrial integrity/cell viability

Cell viability following treatment with dbcAMP ± SP-10 was assessed using the mitochondrial integrity assay [3-(4,5-dimethylthiazol-2-yl)-2,5-diphenyl tetrazolium bromide (MTT)] [20]. 

### 5.6. Real-time quantitative PCR (Q-PCR)

Y-1 cells, cultured in 6-well plates (2 x 10^5^cells/well), were treated with increasing concentrations of SP-10 for the indicated time periods. After treatment, cells were stimulated with 1 mM dbcAMP for 24 h. At the end of the incubation period, total cellular RNA was extracted using RNA STAT-60 according to the manufacturer’s instructions. *hmgcr*, *Tspo* and *Star* mRNAs were quantified by Q-PCR using the ABI Prism 7700 sequence detection system (Perkin-Elmer/Applied Biosystems, Foster City, CA). The primers were designed according to GenBank Accession Numbers using PE/AB Primer Express software, which is specifically designed for the selection of primers and probes. For *hmgcr*, the forward primer was 5'- CCAAGGTGGTGAGAGAGGTGTT-3' (22 nucleotides), and the reverse primer was 5'-CGTCAACCATAGCTTCCGTAGTT-3' (23 nucleotides [20]. For PBR, the forward primer was 5'-CCGCTTGCTGTACCCTTACC-3’ (20 nucleotides), and the reverse primer was 5'-TTGAGCACGGTGGCAAAAG-3’ (19 nucleotides). For StAR, the forward primer was 5'-CTCACAGGAAGCCTGCAAGTC-3’ (21 nucleotides), and the reverse primer was 5'- CCTCCCGATGCTGTTAGCTG-3’ (20 nucleotides) [49]. The primers were synthesized by Bio-Synthesis Inc. (Lewisville, TX). The threshold cycle (Ct) values for both 18S RNA and the samples were calculated using the PE/AB computer software. Ct was determined at the most exponential phase of the reaction. Relative transcript levels were calculated as x = 2^∆∆Ct^, where ∆∆Ct = ∆E - ∆C, ∆E = Ct _experiment_ – Ct _18S_, and ∆C = Ct _control_ – Ct _18s_ [20].

### 5.7. Radioligand-binding assays

[^3^H]PK11195 [1-(2-chlorophenyl)-*N*-methyl-*N*-(1-methylpropyl)-3-isoquinoline carboxamide] (specific activity, 85.5 Ci/mmol) was obtained from NEN Life Science Products (Boston, MA, USA). Binding of [^3^H]PK11195 to 5 μg cell extract was performed as previously described [50]. Specific [^3^H]PK11195 binding was analyzed using the iterative, non-linear, curve-fitting program, RadLig 4.0 (KELL suite, Biosoft, Cambridge, UK). 

### 5.8. Immunoblotting

Y-1 cells were cultured in 6-well plates and treated with dbcAMP ± SP-10 as described for Q-PCR analysis. Cells were washed twice with PBS and lysed in buffer containing 10 mM Tris-HCl, pH 7.5, 150 mM NaCl, 1 mM EDTA, 1 mM EGTA, 1% NP40, and a protease inhibitor cocktail. Samples were prepared as previously described [20]. Proteins were separated by sodium dodecyl sulfate polyacrylamide gen electrophoresis (SDS-PAGE) (4–20% gradient) and transferred onto nitrocellulose membranes. Membranes were blocked and incubated with anti-P450scc antibody (1:800), anti-SR-BI receptor antibody (1:200), or anti-α-actin antibody (1:200). The appropriate peroxidase-conjugated secondary antibodies (Santa Cruz Biotechnology, Santa Cruz, CA, USA) were then applied. Bands were visualized by enhanced chemiluminescence (Amersham Life, Arlington Heights, IL, USA). Densitometry was performed using the Kodak 1D 3.6 analysis software. 

### 5.9. NBD-cholesterol uptake

Y-1 cells plated on 96-well culture plates were treated with SP-10 for 48 h. Cultures then received fresh media containing 1μg/mL NBD-cholesterol ± 1mM dbcAMP plus for 24h. NBD-cholesterol was added from a 1-mg/mL stock solution in ethanol and the ethanol concentration did not exceed 0.1%. To measure cholesterol uptake, cells were washed twice with ice-cold Hanks' balanced salt solution (Biosource, Rockville, MD, USA). Plates were read using the Wallac 1420 Victor^2^ multilabel counter system (PerkinElmer Life Sciences Inc., Boston, MA, USA), equipped with 485-nm excitation and 535-nm emission filters [51]. After measurements were made, cellular proteins were digested with 0.4 N NaOH for 2 h and protein concentrations were determined using the Bio-Red protein Assay kit (Bio-Red Laboratories, Hercules, CA, USA). Bovine serum albumin was used as a standard. Fluorescence values were normalized to total cellular protein content. 

### 5.10. Confocal laser scanning microscopy

Y-1 cell grown on coverslips were treated with SP-10 for 48 h, washed with PBS, and fixed in 4% paraformaldehyde. Cell membranes were permeabilized with 0.1% Triton X-100 prior to a 20 min incubation with Alexa Fluor 488 phalloidin and Alexa Fluor 594 Deoxyribonuclease I to label F-actin and G-actin, respectively. Coverslips were mounted with VECTASHIELD mounting medium containing 4'-6-diamidino-2-phenylindole hydrochloride (DAPI) (Vector Laboratories, Burlingame, CA, USA). Confocal images were acquired using an Olympus Fluoview BX61 laser scanning microscope. Image analysis was performed using the NIH ImageJ software (National Institutes of Health, Bethesda, MD, USA; http://rsb.info.nih.gov/ij/).

### 5.11. Statistics

Statistical analyses were performed using one-way analysis of variance (ANOVA) and Scheffe’s *F* test using JMP IN Version 5 (release 5.1) from Brooks/Cole (Belmont, CA, USA). Values of *p* < 0.05 were accepted as significant. 

## References

[1] McEwen B.S. (1994). Corticosteroids and hippocampal plasticity. Ann. N. Y. Acad. Sci..

[2] Chrousos G.P., Gold P.W. (1992). The concepts of stress and stress system disorders. Overview of physical and behavioral homeostasis. JAMA.

[3] Kiraly S.J., Ancill R.J., Dimitrova G. (1997). The relationship of endogenous cortisol to psychiatric disorder: a review. Can. J. Psychiatry.

[4] Tafet G.E., Toister-Achituv M., Shinitzky M. (2001). Enhancement of serotonin uptake by cortisol: a possible link between stress and depression. Cogn. Affect. Behav. Neurosci..

[5] Maeda K., Tanomoto K., Terada T., Shintani T., Kakigi T. (1991). Elevated urinary free cortisol in patients with dementia. Neurobiol. Aging.

[6] Polleri A., Gianelli M.V., Murialdo G. (2002). Dementia: a neuroendocrine perspective. J. Endocrinol. Invest..

[7] Wolkowitz O.M., Lupien S.J., Bigler E., Levin R.B., Canick J. (2004). The "steroid dementia syndrome": an unrecognized complication of glucocorticoid treatment. Ann. N. Y. Acad. Sci..

[8] Swaab D.F., Raadsheer F.C., Endert E., Hofman M.A., Kamphorst W., Ravid R. (1994). Increased cortisol levels in aging and Alzheimer’s disease in postmortem cerebrospinal fluid. J. Neuroendocrinol..

[9] O’Brien J.T., Ames D., Schweitzer I., Mastwyk M., Colman P. (1996). Enhanced adrenal sensitivity to adrenocorticotrophic hormone (ACTH) is evidence of HPA axis hyperactivity in Alzheimer’s disease. Psychol. Med..

[10] Weiner M.F., Vobach S., Olsson K., Svetlik D., Risser R.C. (1997). Cortisol secretion and Alzheimer’s disease progression. Biol. Psychiatry.

[11] Giubilei F., Patacchioli F.R., Antonini G., Sepe Monti M., Tisei P., Bastianello S., Monnazzi P., Angelucci L. (2001). Altered circadian cortisol secretion in Alzheimer’s disease: Clinical and neuroradiological aspects. J. Neurosci. Res..

[12] Rasmuson S., Nasman B., Carlstrom K., Olsson T. (2002). Increased levels of adrenocortical and gonadal hormones in mild to moderate Alzheimer’s disease. Dement. Geriatr. Cogn. Disord..

[13] Corley P.A. (1996). Acquired immune deficiency syndrome: The glucocorticoid solution. Med. Hypotheses.

[14] Bhansali A., Dash R.J., Sud A., Bhadada S., Sehgal S., Sharma B.R. (2000). A preliminary report on basal and stimulated plasma cortisol patients with acquired immunodeficiency syndrome. Indian J. Med. Res..

[15] Christeff N., Nunez E.A., Gougeon M.L. (2000). Changes in cortisol/DHEA ratio in HIV-infected men are related to immunological and metabolic perturbations leading to malnutrition and lipodystrophy. Ann. N. Y. Acad. Sci..

[16] Erkut Z.A., Endert E., Huitinga I., Swaab D.F. (2002). Cortisol is increased in postmortem cerebrospinal fluid of multiple sclerosis patients: relationship with cytokines and sepsis. Mult. Scler..

[17] Luecken L.J., Compas B.E. (2002). Stress, coping, and immune function in breast cancer. Ann. Behav. Med..

[18] Jefcoate C. (2002). High-flux mitochondrial cholesterol trafficking, a specialized function of the adrenal cortex. J. Clin. Invest..

[19] Azha r S., Leers-Sucheta S., Reaven E. (2003). Cholesterol uptake in adrenal and gonadal tissues: the SR-BI and ‘selective’ pathway connection. Front. Biosci..

[20] Xu J., Lecanu L., Han Z., Yao Z., Greeson J., Papadopoulos V. (2003). Inhibition of adrenal cortical steroid formation by procaine is mediated by reduction of the cAMP-induced 3-hydroxy-3-methylglutaryl-coenzyme A reductase messenger ribonucleic acid levels. J. Pharmacol. Exp. Ther..

[21] Xu J., Lecanu L., Tan M., Yao W., Greeson J., Papadopoulos V. (2007). The benzamide derivative N-[2-(4-cyclopropanecarbonyl-3-methyl-piperazin-1-yl)-1-(1H-indol-3-yl-methyl)-2-oxo-ethyl]-4-nitro-benzamide (SP-10) reduces HIV 1 infectivity *in vitro* by modifying actin dynamics. Antivir. Chem. Chemother..

[22] Papadopoulos V., Baraldi M., Guilarte T.R., Knudsen T.B., Lacapère J.J., Lindemann P., Norenberg M.D., Nutt D., Weizman A., Zhang M.R., Gavish M. (2006). Translocator protein (18kDa): new nomenclature for the peripheral-type benzodiazepine receptor based on its structure and molecular function. Trends Pharmacol. Sci..

[23] Stocco D.M. (2001). StAR protein and the regulation of steroid hormone biosynthesis. Annu. Rev. Physiol..

[24] Atshaves B.P., Starodub O., McIntosh A., Petrescu A., Roths J.B., Kier A.B., Schroeder F. (2000). Sterol carrier protein-2 alters high density lipoprotein-mediated cholesterol efflux. J. Biol. Chem..

[25] Frolov A., Petrescu A., Atshaves B.P., So P.T.C., Gratton E., Serrero G., Schroeder F. (2000). High density lipoprotein-mediated cholesterol uptake and targeting to lipid droplets in intact L-cell fibroblasts. A single- and multiphoton fluorescence approach. J. Biol. Chem..

[26] Capponi A.M. (2002). Regulation of cholesterol supply for mineralocorticoid biosynthesis. Trends Endocrinol. Metab..

[27] Estes J.E., Selden L.A., Gershman L.C. (1981). Mechanism of action of phalloidin on the polymerization of muscle actin. Biochemistry.

[28] Howard T.H., Oresajo C.O. (1985). The kinetics of chemotactic peptide-induced change in F-actin content, F-actin distribution, and the shape of neutrophils. J. Cell. Biol..

[29] Cassimeris L., McNeill H., Zigmond S.H. (1990). Chemoattractant-stimulated polymorphonuclear leukocytes contain two populations of actin filaments that differ in their spatial distributions and relative stabilities. J. Cell. Biol..

[30] Brown A.S., Hall P.F., Shoyab M., Papadopoulos V. (1992). Endozepine/Diazepam Binding Inhibitor in adrenocortical and Leydig cell lines: Absence of hormonal regulation. Mol. Cell. Endocr..

[31] Lecanu L., Greeson J., Papadopoulos V. (2006). Beta-amyloid and oxidative stress jointly induce neuronal death, amyloid deposits, gliosis, and memory impairment in the rat brain. Pharmacology.

[32] Ferrari E., Fioravanti M., Magri F., Solerte S.B. (2000). Variability of Interactions Between Neuroendocrine and Immunological Functions in Physiological Aging and Dementia of the Alzheimer's Type. Ann. N. Y. Acad. Sci..

[33] Simpson E., Lauber M., Demeter M., Stirling D., Rodgers R., Means G., Mahendroo M., Kilgore M., Mendelson C., Waterman M. (1991). Regulation of the genes encoding steroidogenic enzymes. J. Steroid Biochem. Mol. Biol..

[34] Connelly M.A., Williams D.L. (2003). SR-BI and cholesterol uptake into steroidogenic cells. Trends in Endo. Metab..

[35] Plump A.S., Erickson S.K., Weng W., Partin J.S., Breslow J.L., Williams D.L. (1996). Apolipoprotein A-I is required for cholesteryl ester accumulation in steroidogenic cells and for normal adrenal steroid production. J. Clin. Invest..

[36] Liu J., Heikkila P., Meng Q.H., Kahri A.I., Tikkanen M.J., Voutilainen R. (2000). Expression of low and high density lipoprotein receptor genes in human adrenals. Eur. J. Endocrinol..

[37] Landschulz K., Pathak R.K., Rigotti A., Krieger M., Hobbs H.H. (1996). Regulation of scavenger receptor, class B, Type I, a high density lipoprotein receptor, in liver and steroidogenic tissues of the rat. J. Clin. Invest..

[38] Azhar S., Nomoto A., Reaven E. (2002). Hormonal regulation of adrenal microvillar channel formation. J. Lipid Res..

[39] Fu T., Kozarsky K.F., Borensztajn J. (2006). Overexpression of SR-BI by adenoviral vector reverses the fibrate-induced hypercholesterolemia of apolipoprotein E-deficient mice. J. Biol. Chem..

[40] Mardones P., Pilon A., Bouly M., Duran D., Nishimoto T., Arai H., Kozarsky K.F., Altayo M., Miquel J.F., Luc G., Clavey V., Staels B., Rigotti A. (2003). Fibrates down-regulate hepatic scavenger receptor class B type I protein expression in mice. J. Biol. Chem..

[41] Briand O., Lestavel S., Pilon A. (2003). SR-BI does not require raft/caveola localization for cholesteryl ester selective uptake in the human adrenal cell line NCI-H295R. Biochim. Biophys. Acta.

[42] Reaven E., Cortez Y., Leers-Sucheta S., Nomoto A., Azhar S. (2004). Dimerization of the scavenger receptor class B type I: formation, function, and localization in diverse cells and tissues. J. Lipid Res..

[43] Revenu C., Athman R., Robine S., Louvard D. (2004). The co-workers of actin filaments: From cell structures to signals. Nat. Rev. Mol. Cell. Biol..

[44] Deng Y.L., Utsunomiya H., Osamura Y. (1990). The role of actin in changes of cell shape and steroidogenesis in mouse Y-1 cells stimulated by ACTH--immunocytochemical studies. Tokai J. Exp. Clin. Med..

[45] Hall P.F. (1985). On the mechanism of action of ACTH: The role of actin. Endocr. Res..

[46] Loesser K.E., Malamed S. (1987). A morphometric analysis of adrenocortical actin localized by immunoelectron microscopy: The effect of adrenocorticotropin. Endocrinology.

[47] Loesser K.E., Cain L.D., Malamed S. (1994). The peripheral cytoplasm of adrenocortical cells: Zone-specific responses to ACTH. Anat. Rec..

[48] Hall P.F. (1995). The roles of microfilaments and intermediate filaments in the regulation of steroid synthesis. J. Steroid Biochem. Mol. Biol..

[49] Li W., Amri H., Huang H., Wu C., Papadopoulos V. (2004). Gene and protein profiling of the response of MA-10 Leydig tumor cells to human chorionic gonadotropin. J. Androl..

[50] Papadopoulos V., Amri H., Li H., Boujrad N., Vidic B., Garnier M. (1997). Targeted disruption of the peripheral-type benzodiazepine receptor gene inhibits steroidogenesis in the R2C Leydig tumor cell line. J. Biol. Chem..

[51] Lada A.T., Davis M., Kent C., Chapman J., Tomoda H., Omura S., Rudel L.L. (2004). Identification of ACAT1- and ACAT2-specific inhibitors using a novel, cell-based fluorescence assay: individual ACAT uniqueness. J. Lipid Res..

[52] Sapolsky R.M. (2000). Glucocorticoids and hippocampal atrophy in neuropsychiatric disorders. Arch. Gen. Psychiatry.

[53] Dedovic K., Duchesne A., Andrews J., Engert V., Pruessner J.C. (2009). The brain and the stress axis: The neural correlates of cortisol regulation in response to stress. NeuroImage.

